# Effects of selenium supplementation on glucose homeostasis in women with gestational diabetes mellitus: A randomized, controlled trial

**DOI:** 10.18502/ijrm.v18i1.6201

**Published:** 2020-01-27

**Authors:** Fatemeh Sadat Najib, Tahereh Poordast, Mina Rezvan Nia, Mohammad Hossein Dabbaghmanesh

**Affiliations:** ^1^Infertility Research Centere of Obstetrics and Gynecology Ward, Shiraz Medical School, Shiraz University of Medical Sciences, Shiraz, Iran.; ^2^Endocrin and Metabolic Research center of Internal Medicine Ward, Shiraz Medical School, Shiraz University of Medical Sciences, Shiraz, Iran.

**Keywords:** Diabetes, Gestational, Selenium.

## Abstract

**Background:**

There is limited evidence about the anti-diabetic effects of selenium supplementation in women with gestational diabetes mellitus (GDM).

**Objective:**

This study investigates the effects of selenium supplementation on glucose homeostasis in women with GDM.

**Materials and Methods:**

A total of 60 pregnant women with GDM were enrolled in this prospective randomized, double-blind, and placebo-controlled clinical trial. They were randomly assigned to take either 100µg selenium supplements as tablet or a placebo daily for 12 wk since 24-28 wk of gestation. The primary outcomes were changes in the glucose homeostasis, including fasting plasma glucose, the 2-hr post prandial blood glucose, serum insulin level, glycosylated hemoglobin (Hb A1C), and the homeostasis model assessment of insulin resistance(HOMA_IR) at the initial period and 3 months after intervention.

**Results:**

The mean maternal age of the patients who took selenium supplements was 29.19 ± 6.16 (range 18-41) years. In the placebo group, the mean maternal age was 31 ± 4.43 (range 24-39) years. Compared with the placebo group, fasting plasma glucose, 2-hr post-prandial blood glucose, glycosylated hemoglobin(Hb A1C), serum insulin level, and homeostasis model of assessment-estimated insulin resistance(HOMA_IR) were not significantly changed in the selenium group at the end of study (p = 0.25, p = 0.87, p = 0.34, p = 0.57, and p = 0.31, respectively).

**Conclusion:**

The results of this trial suggest that supplementation with 100µg of selenium does not modulate glucose homeostasis in women with GDM.

## 1. Introduction

Gestational diabetes mellitus (GDM) is a temporary form of type 2 diabetes mellitus (T2DM) and is one of the major health problems affecting pregnant women (1). In fact, that is impaired insulin metabolism and carbohydrate intolerance with differing levels of severity for first time recognition during pregnancy (2). In a normal pregnancy with an increase in oxidative stress, insulin resistance increases (3) and insulin secretion was decreased, thus linking it to T2DM in some mothers (4).

Selenium, a dietary supplement, with antioxidant action, which is required for the activity of glutathione peroxidase (5). Dietary sources of selenium is nuts, cereals, meat, mushrooms, fish, and eggs (6). Being an essential element of the enzymes, selenium protects cells from the deleterious impact of free radicals (7, 8). Recent evidence support the effective role of selenium on hypertension (9), coronary artery disease (10), some cancers (11), and inflammatory diseases (12). Selenium may be involved in reducing the severity of insulin resistance in diabetes (13). Therefore, it appears to bear anti-diabetic functions emerging from its insulin-like properties; accordingly, selenium supplementation in diabetic patient is likely to have beneficial effects on glocuse utilization (14).

There are controversial results about the association between selenium status and GDM. Some investigations have proved pregnant women with DM showing lower concentrations of serum selenium in comparison with their healthy counterparts (15). However, evidenced by Molnar *et al*. (16), serum selenium concentration is conspicuously higher in pregnant women with DM. In another query, Al-Saleh *et al*. (17) failed to detect a significant relationship between serum selenium concentration and GDM. A recent clinical trial evaluated the effects of selenium supplementation on the metabolic profile of women with GDM (18). The results demonstrated a considerable reduction in the fasting plasma glucose (FPG), serum insulin level, homeostasis model assessment of insulin resistance (HOMA-IR).

Because of limited evidence about the anti-diabetic effects of selenium supplementation in women with GDM, our study was designed to investigate the effects of selenium supplementation on glucose homeostasis in pregnant women with GDM.

## 2. Materials and Methods

### Trial design

This study was designed as a prospective randomized, double-blind, placebo-controlled, clinical trial which was conducted on pregnant women with GDM who were referred to the outpatient clinic of Hafez and Zeinabieh hospital (Shiraz, Iran) during a 16 month period from December 2015 to March 2017.

### Participants

A total of 60 consecutive pregnant women aged between 18 and 40 yr with a diagnosis of GDM by the 75-g oral glucose tolerance test (OGTT) at 24 to 28-wk gestation who were carrying singleton pregnancies were enrolled in the study. The exclusion criteria included patients who require substitute treatments, including hormones, patients on oral hypoglycemic agents (OHAs), and those who require insulin therapy. Patients with hypo- or hyperthyroidism and smokers were also excluded from the study. GDM was diagnosed on the basis of the criteria set by the American Diabetes Association (19). Specifically, those whose plasma glucose meets one of the following criteria were considered to have GDM: fasting ≥92 mg/dL, 1-hr after 75 gr glucose ≥180 mg/dL, or 2-hr after 75 gr glucose ≥153 mg/dL.

### Interventions and outcomes

Women were randomly assigned to take either 100 µg selenium supplements as tablet or a placebo daily for 12 wk beginning from the 24${}^{\mathrm{th}}$ to the 28${}^{\mathrm{th}}$ wk of gestation. The shape and packing of both tablets were similar to ensure double-blind design. The prescribed selenium dose was in accordance with the National Academy of Sciences guidelines that established an upper limit of 400 mg/d of selenium (20, 21). The selenium and the matched placebo tablets were purchased from pharma Nord company (Vejle, Denmark). The placebo tablets contained Avicel 102 (microcrystalline cellulose). Both groups were co-administered under an anti-diabetic diet by a nutritionist and were recommended to do light exercises.

We evaluated the patients' fasting plasma glucose (FPG) every 3 wk during the study period to determine if any required insulin therapy so as to exclude such patients from the study.

The baseline characteristics of the participants, including maternal age, gestational age, height, weight, and BMI were obtained by a data gathering form. The main outcomes were the glucose homeostasis changes including FPG, 2-hr post prandial glucose (2HPPG) test with an enzymatic method (glucose oxidase and peroxidase kits: pars azmoon), serum insulin level by ECL method (electrochemiluminescene kit: Immulit 2000), HbA1c with the CERAGEM method (kit: cerastat 2000), HOMA-IR at the initial period and 3 months after the intervention.

HOMA-IR was estimated with the formula below: 

 HOMA − IR = Glucose  mg  dl × fasting  insulin  mIU  mL 405

Healthy range: 1.0 (0.5-1.4); Less than 1.0: Insulin-sensitive which is optimal; Above 1.9: Early insulin resistance; Above 2.9: significant insulin resistance.

### Randomization

Using a randomization table, randomization was carried out based on the patient registration number. Then blinded and labeled with a four-digit code, study pills were packaged in separate packs. The project coordinator maintained the information in terms of the codes relating to a particular treatment. All the patients, attending physicians, staff involved in the study, and members garnering and analyzing data were blinded to the intervention other than the project coordinator.

### Ethical consideration

The study protocol was approved by the Institutional Review Board of SUMS and we obtained Ethical Approval from the Local ethics Committee (IR.SUMS.MED.REC.1395.13) before the study was commenced. All the participants gave their informed written consent.

### Statistical analysis

The number of the samples used in this study is based on data from previous studies and the use of the MedCak software. A 90% power and a 5% error were estimated for 27 persons in each group. To shun the consequences of possible attrition and exclusion from the study, 30 participants were enrolled in each group. Statistical analyses were conducted deploying the Statistical Package for Social Sciences version 19.0 (SPSS Inc., Chicago, IL, USA). Kolmogorov-Smirnov test was employed to analyze the distribution of the variable. The Chi-square (χ2) test was also deployed to analyze nominal variables. Normally-distributed Kolmogorov-Smirnov test parametric variables were tested by independent Student's t-test. Non-normally-distributed metric variables were also analyzed by Mann-Whitney U test. Data were then reported as means ± SD. A two-sided p-value of less than 0.05 was considered statistically significant.

## 3. Results

### Baseline characteristics

**Figure 1:**
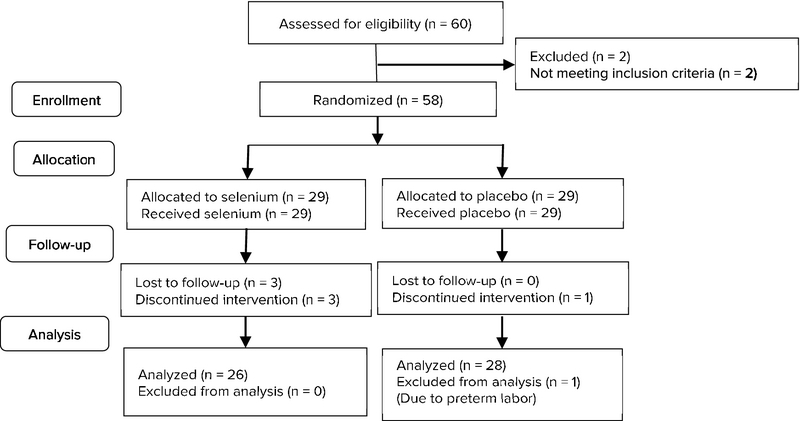
Consort 2010 flow diagram.

Out of the 60 patients that were randomized into the two study groups, six patients were excluded. Four women from the selenium group were excluded: one because of insulin therapy while three were lost to follow-up. Two women in the placebo group were excluded: one for preterm labor pregnancy and one because OF high blood sugar, she required insulin therapy. Ultimately, 26 patients were enrolled in the selenium group and 28 individuals were enrolled in the placebo group. Figure 1 shows the Consort flow diagram of this trial.

The baseline characteristics of the contributors are shown in Table I, There were no significant differences between the groups at baseline. The mean maternal age, gestational age, and BMI of the patients who consumed selenium and the placebo group were the same. There were no significant differences between the study groups regarding gravidity and history of previous abortions. Also, the number of gravida and history of abortion among the participants in the selenium and placebo groups are the same. In addition, there were no differences in the parameters of glucose homeostasis between the study groups at baseline.

### Outcomes of glucose homeostasis

**Figure 2:**
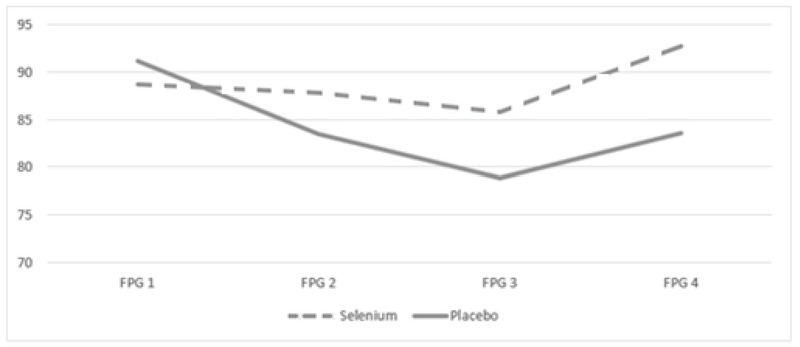
The changing flow of FPG for each group.

Compared with the placebo group, FPG, 2HPPG, HbA1C, serum insulin, and HOMA-IR were not significantly changed in the selenium group at the end of study (p = 0.25, p = 0.87, p = 0.34, p = 0.57, and p = 0.31, respectively) (Table II). In addition, we evaluated the patients by FPG every 3 wk during the study period. However, we did not find any significant differences regarding FPG between the study groups with the 3-wk intervals. Figure 2 shows the changing flow of FPG for each group.

In another analysis, we compared the changes in the parameters of glucose homeostasis from baseline between the groups. All of the parameters decreased after 3 months in the two study groups but in comparing these changes, they were not significant (Table III). Finally, we did not find any significant effect of taking selenium supplements on FPG, 2HPPG, HbA1C, serum insulin, and HOMA-IR.

**Table 1:** Baseline characteristics of the participants


	**Selenium group (n = 26)**	**Placebo group (n =28)**	**p-value**
Maternal age (years)*a	29.19 ± 6.16	31 ± 4.43	0.09
Gestational age (weeks)*a	27.15 ± 2.16	26.52 ± 1.91	0.31
Gravidity${}^{\#}$b		0.29
Primigravida	11 (42.3)	8 (28.6)	
Multigravida	15 (57.7)	20 (71.4)	
History of previous abortion${}^{\#}$ b	5 (19.2)	6 (21.4)	0.84
BMI (kg/m${}^{2}$)*a	28.55 ± 3.76	28.03 ± 3.38	0.59
Glucose homeostasis*a
FPG (mg/dL)	88.73 ± 11.95	91.21 ± 8.21	0.37
OGTT-1hr (mg/dL)	186.15 ± 24.92	190.07 ± 20.85	0.53
OGTT-2hr (mg/dL)	149.15 ± 37.07	150.42 ± 16.84	0.87
HbA1c (%)	5.55 ± 0.59	5.34 ± 0.69	0.24
Serum Insulin (mIU/mL)	17.09 ± 6.91	15.15 ± 5.77	0.26
HOMA-IR	3.83 ± 1.71	3.29 ± 1.49	0.22
a: Data presented as Mean ± SD		b: Data presented as n (%)	
P<0.05 was considered significant		*Student t-test	
# Chi-square (χ2) test		FPG: Fasting plasma glucose	
OGTT: Oral glucose tolerance test			
HOMA-IR: Homeostasis model of assessment-estimated insulin resistance			

**Table 2:** Comparing glucose homeostasis variables between two study groups at the end of the study


**Glucose homeostasis**	**Selenium group (n = 26)**	**Placebo group (n = 28)**	**p-value**
FPG (mg/dL)${}^{*}$	92.75 ± 9.21	83.51 ± 2.12	0.25
2HPPG (mg/dL)${}^{\$}$	136.21 ± 13.97	137.42 ± 24.04	0.87
HbA1c (%)${}^{\$}$	5.35 ± 0.54	5.31 ± 0.55	0.34
Serum insulin (mIU/mL)${}^{\$}$	15.96 ± 5.63	15.13 ± 4.89	0.57
HOMA-IR${}^{\$}$	3.37 ± 1.27	3.04 ± 1.09	0.31
Data presented as Mean ± SD		A p-value of <0.05 was significant	
*Student's t-test		$Mann-Whitney U test	
FPG: Fasting plasma glucose		2HPPG: 2-hr post prandial glucose test	
HOMA-IR: Homeostasis model assessment of insulin resistance			

**Table 3:** Comparing changes in glucose homeostasis parameters from baseline to 3 months


**Glucose homeostasis**	**Selenium group (n = 26)**	**Placebo group (n = 28)**	**p-value**
FPG (mg/dL)	3.51 ± 1.22	-3.52 ± 3.53	0.49
2HPPG (mg/dL)	-12.96 ± 8.91	-13 ± 7.54	0.95
HbA1c (%)	-0.21 ± 0.43	-0.14 ± 0.25	0.51
Serum insulin (mIU/mL)	-1.13 ± 3.81	-0.01 ± 2.58	0.33
HOMA-IR	-0.45 ± 1.15	-0.25 ± 1.13	0.52
Data presented as Mean±SD		A p-value <0.05 was significant (Mann-Whitney U test)	
FPG: Fasting plasma glucose		2HPPG: 2-hr post prandial glucose test	
HOMA-IR: Homeostasis model assessment of insulin resistance			

## 4. Discussion

Selenium is known as an essential trace element bearing antioxidant activity as well as anti-inflammatory effects (22). Decrease concentrations of selenium in whole blood and plasma during pregnancy is significant compared with pre-pregnancy or non-pregnancy concentrations; with the progression of gestation, this decrease in selenium continues (23). Because of fetal growth, there is a demand for selenium during pregnancy, which projects as lowering maternal selenium concentrations. These reductions are higher in pregnant women suffering from gestational diabetes (14, 24). In spite of such findings, few clinical trials have investigated the efficacy of selenium supplementation in controlling glucose homeostasis in GDM. The results of our trial suggest that taking 100µg selenium supplement did not have significant effects on the FPG, 2HPPG, HbA1C, serum insulin, and HOMA-IR of pregnant women with GDM.

To the best of our knowledge, there has been one report about the role of selenium supplementation in GDM. Identified by their method, Asemi *et al*. randomly categorized their patients to administer either 200μg selenium supplements (n = 35) or a placebo (n = 35) for 6 wk from wk 24 to wk 28 of gestation to their patients (18). Their results demonstrated that selenium supplementation in pregnant women with GDM had a positive role in improving glucose homeostasis, reducing inflammation, and improving oxidative stress. However, it did not affect the lipid profiles or plasma nitric oxide. The results were in contrast with what was found in our study. It is to be noted that in Asemi's *et al* trial, the participants' dietary features and physical activity records were established to ensure maintenance of their routine diet and physical activity during the research (18). Moreover, dietary intakes of energy, carbohydrates, fatty acids, proteins, cholesterol, total dietary fiber, selenium, magnesium, and vitamins C, E, and A were finally compared the results of which displayed no significant differences. In our study, however, participants were demanded to alter their routine physical activity and usual dietary intakes; they were required to consume the supplements only provided by the investigators. Nevertheless, we did not record and compare their dietary intakes. This is an important limitation of this study and may partly explain why the selenium supplementation was ineffective for GDM. Another reason for the difference between our results and that of Asemi *et al* (18) can be attributed to the selenium dose. They administrated 200 μg of selenium, whereas we used 100 μg.

No side effects were fortunately reported following selenium consumption in this study. Interestingly, supplemental selenium dose deployed was lower than the upper limits (400 mg). However, there is controversy as to the toxic impact of selenium on health. Earlier, Burk *et al*. (25) reported an intake of moderate (200 mg/d) to large doses (600 mg/d) of selenium supplements for 16 wk as safe in individuals aged ≥18 years. In another study, no considerable escalation of the diabetes risk following selenium supplementation was identified (26). However, some investigations have enumerated hair loss, dystrophic fingernail, gastrointestinal symptoms, and memory impairment as the adverse effects stemming from selenium intake (27). Moreover, some studies demonstrate high selenium intake likely having toxic effects on growth hormone levels, insulin-like growth factor-1, and thyroid function (28). Further queries are accordingly required to appraise the potential toxicity/teratogenicity of selenium supplements intake in the long run.

##  Limitations

There are some limitations associated with the present study. A major limitation is the relatively few participants enrolled to observe the differences in the primary endpoints. Therefore, the conclusion can hardly be avoided as to the need for validation of the relative effects of selenium therapy through large-scale investigations. Another limitation lies in not controlling the impact of selenium supplementation on lipid profiles, liver enzymes and kidney function as well as on selenium-dependent antioxidant enzymes such as GPx isoforms and thioredoxin reductase. Additionally, as mentioned earlier, we did not record and compare the dietary intakes of the patients.

## 5. Conclusion

In summary, the results of this trial suggest that supplementation with 100µg selenium has no effect on glucose homeostasis in women with GDM.

##  Conflicts of Interest

None of the co-authors have any financial or another relevant conflict of interest in the material covered by the manuscript.
